# The Vascular Microenvironment and Systemic Sclerosis

**DOI:** 10.1155/2010/362868

**Published:** 2010-08-10

**Authors:** Tracy Frech, Nathan Hatton, Boaz Markewitz, Mary Beth Scholand, Richard Cawthon, Amit Patel, Allen Sawitzke

**Affiliations:** ^1^Division of Rheumatology, Department of Internal Medicine, University of Utah, Salt Lake City, UT 84132, USA; ^2^Division of Respiratory, Critical Care and Occupational Pulmonary Medicine, Department of Internal Medicine, University of Utah, Salt Lake City, UT 84132, USA; ^3^Department of Human Genetics, University of Utah, Salt Lake City, UT 84132, USA; ^4^Department of Cardiothoracic Surgery, University of Utah, Salt Lake City, UT 84132, USA

## Abstract

The role of the vascular microenvironment in the pathogenesis Systemic Sclerosis (SSc) is appreciated clinically as Raynaud's syndrome with capillary nail bed change. This manifestation of vasculopathy is used diagnostically in both limited and diffuse cutaneous subsets of SSc, and is thought to precede fibrosis. The degree of subsequent fibrosis may also be determined by the vascular microenvironment. This paper describes why the vascular microenvironment might determine the degree of end-organ damage that occurs in SSc, with a focus on vascular cell senescence, endothelial progenitor cells (EPC) including multipotential mesenchymal stem cells (MSC), pericytes, and angiogenic monocytes. An explanation of the role of EPC, pericytes, and angiogenic monocytes is important to an understanding of SSc pathogenesis. An evolving understanding of the vascular microenvironment in SSc may allow directed treatment.

## 1. Introduction

Systemic sclerosis (SSc, scleroderma) is an autoimmune disease characterized early in the process by vasculopathy and subsequently by varying degrees of fibrosis in skin, lungs, and other tissues. The presence of vasculopathy is the hallmark of this condition, represented clinically as Raynaud's syndrome which occurs almost universally in both the limited and diffuse cutaneous subsets of this disease. Calcinosis and telangiectasias are also features of SSc vascular damage. Vasculopathy possibly results from abnormal vasoreactivity, hypoxia, and/or direct damage of vascular and perivascular cells [1]. Perivascular inflammatory infiltrates and neoangiogenesis ensues resulting in varying degrees of fibrosis in the skin and internal organs [2]. This paper describes why details of the vascular microenvironment might determine the degree of end-organ damage that occurs in SSc, with a focus on vascular cell senescence, endothelial progenitor cells (EPC) including mesenchymal stem cells (MSC), pericytes, and angiogenic monocytes. An explanation of the role of EPC, pericytes, and angiogenic monocytes is important to understanding SSc pathogenesis. 

SSc is thought to be a genetically complex disease, influenced by multiple genes, with a substantial environmental component [3]. Nonetheless, SSc occurs significantly more frequently in families with scleroderma (1.6%) than in the general population (0.026%) [4]. Genome-wide association studies have found a strong association with the HLA II region on chromosome 6, and non-HLA candidate genes that regulate interferon production, such as interferon regulatory factor 5 (IRF 5) as well as genes that regulate immunological responses, such as signal transducer and activator 4 (STAT 4) [5, 6]. There are also multiple HLA class II associations with autoantibody markers and subphenotypes [7]. As such, systemic sclerosis is an autoimmune disease; however the inherited effects of vasculopathy and fibrosis remain to be determined. Our previous work showed that vasculopathy imparts a greater relative risk to family members than does autoimmune inflammatory conditions or fibrotic lung disease [8].

## 2. Vascular Senescence

The microvascular environment in SSc has a reduced density and disorganized structure [9]. Irrespective of the subset of SSc, perivesicular inflammatory infiltrates result in endothelial derangement in lesioned as well as perilesional tissue [10, 11]. These perivascular changes precede the excessive accumulation of extracellular matrix components, and fibrosis may represent a default pathway from vascular failure [12, 13]. The histopathological hallmark in SSc is a result of endothelium activation with cell adhesion molecule expression, inflammatory cell recruitment, intimal proliferation, and adventitial fibrosis, which results in apoptosis of endothelial cells [13, 14]. Despite the ensuing severe tissue hypoxia, proper adapted angiogenesis does not occur in SSc [2].

Vascular cells normally have a finite lifespan which is determined in part by telomere length and/or telomerase activity [15]. Telomerase is a reverse transcriptase which helps maintain telomere length, thereby preventing cell senescence and protecting chromosomes from aberration. Although telomerase activity is increased in many connective tissue diseases, it is decreased in systemic sclerosis (SSc), perhaps due to gene polymorphism [16, 17].

**Figure 1:**
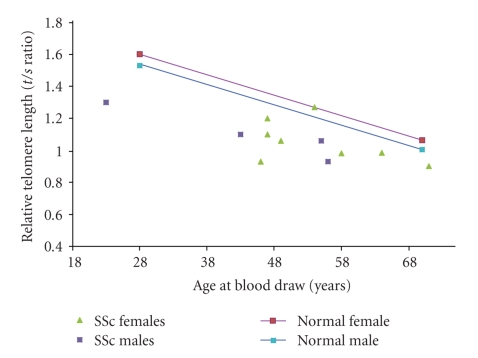
Telomere length of females and males with Systemic Sclerosis (SSc) compared to Healthy Controls.

**Figure 2:**
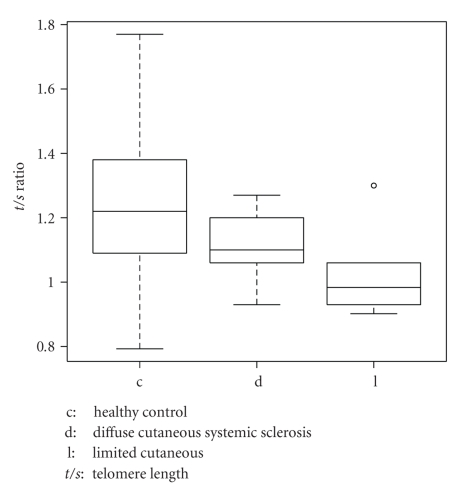
Telomere length of Healthy Controls, diffuse cutaneous Systemic Sclerosis, limited cutaneous Systemic Sclerosis.

There have been contrasting reports of telomere length in SSc. Artlett and colleagues reported a decrease telomere length in a combined cohort of limited SSc (lSSc) and diffuse SSc (dSSc) whereas MacIntyre and colleagues reported increased telomere length and lack of age-related telomere erosion in lSSc [18, 19]. In a pilot study, we used a monochrome multiplex quantitative PCR (MMQPCR) method to evaluate the relative telomere lengths (*t/s* ratios) in DNA samples of 6 lSSc (1 male; 5 females) and 6 dSSc (3 males; 3 females) aging 40–60, and 50 healthy controls (HC) aging 37–60 [20]. Two factors were statistically associated (*P* value <.001) to *t/s*: age and diagnosis (Figure 1). Not correcting for age, the average length measure was 1.2 for normals, 1.15 for dSSc and 0.96 for lSSc patients (Figure 2). Gender was not statistically associated with *t/s*. Telomere length, which is shorter in SSc patients than in normal HC, is possibly a risk factor for vasculopathy. While the appearance of vasculopathy does not vary per subtype of SSc, the effect of telomere length on the fibrocyte or myofibroblast may be different in lSSc and dSSc, possibly contributing to differences in disease manifestations. The reduced telomere length in the endothelial cell likely results in chronic underperfusion and ischemia in the skin and internal organs in both lSSc and dSSc. However, if fibrosis is the default pathway of insufficient angiogenic response, the subsequent reduced lifespan of the fibrocyte (determined by telomere length) may be protective in the lSSc subtype.

## 3. Endothelial Progenitor Cells and Pericytes

The vascular network is a dynamic organ with an estimated surface area of >1000 m^2^ [21]. Neovascularization is a complex process that requires both the mobilization of cells derived from the bone marrow, named endothelial progenitor cells (EPCs), and proliferation and differentiation of resident cells, known as pericytes, to migrate to the correct location and assemble into vascular structures [22]. 

New vessels are produced by a combination of angiogenesis and vasculogenesis. In angiogenesis, fully differentiated endothelial cells arise from pre-existing vessels whereas vasculogenesis describes the formation of new vessels by circulating EPC which act to replenish damaged or senescent blood vessels [14]. This process requires dynamic and temporally regulated interactions between endothelial cells, soluble proangiogenic and antiangiogenic growth factors, and extracellular matrix molecules [23].

Primary contact between endothelial cells and mural cells (pericyte and vascular smooth muscle cells) is central to the regulation of vascular formation in angiogenesis [24]. Recently formed endothelial tubes are initially unstable and become stabilized through the formation of a peri-vascular matrix and the connection with pericytes [25]. Pericytes are embedded within the endothelial basement membrane and are found primarily around blood capillaries, precapillary arterioles, postcapillary venules, and collecting venules [26]. They are arranged to facilitate and assimilate cell communication. With particular interest to SSc, pericytes may play a role in ectopic calcification and are able to transdifferentiate into fibroblast-like cells if they escape from the capillary basement membrane [27]. Furthermore, mural cell defects are reported in other diseases characterized by telangiectasias [28]. The pericyte role as a perivasicular mesenchymal stem cell with macrophage-like properties has not been welldefined in SSc, but is intriguing.

The pericyte is critical for maintenance of vascular stability. Its ability to perform this function is correlated with marker expression and the microenvironment of the endothelial-pericyte contact. Most likely, specific intercellular signals mediated by ligand-receptor systems are required for endothelial and pericyte vascular stability [24]. Numerous studies demonstrate the critical importance of transforming growth factor- (TGF)-beta signaling for vascular development and function [24]. TGF-beta has context-dependent effects on endothelial cells; proliferation is mediated by signaling through ALK/Smad1/5 and differentiation is mediated by ALK Smad2/3 [29]. TGF-beta/Smad signaling has been suggested to play a key role in the pathogenesis of SSc [30]. 

In postnatal vasculogenesis, pericytes develop from tissue-derived stem cells and/or peripheral EPC [31]. Identification and quantification of EPC population in SSc has been challenging and has resulted in consensus recommendations to help unify EPC research [14]. Research by Avouac and colleagues, using an accurate, reliable, and reproducible method of EPC quantification, supports that SSc is associated with EPC mobilization, but in active or severe stages, EPC may be recruited to injured sites and thus decrease in the circulation [9]. 

Multipotential mesenchymal stem cells (MSCs) might be a source of EPC in vasculogenesis [2]. MSCs show normal functional properties and a normal pattern of biological markers, but the angiogenic potential of these endothelial-like MSCs is reduced [32]. Cipriani and colleagues showed that when MSC from SSc patients are seeded on Matrigel, they have a reduced ability to form capillary-like structures and give rise to incomplete endothelial networks, even after vascular endothelial growth factor (VEGF) and stromal-derived factor (SDF-1) stimulation [23, 32].

VEGF is an important angiogenic peptide with specific proliferative, differentiation, and mobilization effects on EPCs, and is known to be upregulated in SSc, especially in advanced disease [33]. VEGF gene expression is also regulated by growth factors (such as TGF-beta) and other proinflammatory cytokines. The platelet-derived growth factor (PDGF) family is essential to vascular remodeling and maturation [34]. In a study of 62 SSc patients, EPCs were significantly increased in patients with early-stage SSc disease, but not in those with late disease irrespective of diagnosis subtype, and there was no correlation between the number of circulating EPCs and VEGF [24]. Bone marrow biopsy samples from 14 of these SSc patients (3 early limited SSc, 4 with late limited SSc, 4 with early diffuse SSC, and 3 with late SSc) showed fewer and functionally impaired EPCs in all patients [33]. Another study showed that the subset of SSc patients with digital lesions and high severity scores had low EPC counts [35]. It is possible that bone marrow from SSc patients cannot satisfy the continuous and prolonged demand for EPCs, despite the target organ increase in VEGF [33]. 

The role of target organ microvascular environment (pericytes and endothelial cells), which is producing TGF-beta, VEGF, and PDGF, on SSc pathogenesis is less clear. TGF-beta can be either pro- or antiangiogenic based on its concentration [36]. The elevated total number and activated state of circulating endothelial cells (CECs), suggest vascular damage and endothelial activation in SSc patients regardless of subtype correlates to disease activity [37]. Thus, vascular damage may drive the disease. It is also known that TGF-beta and PDGF from this microvasculature cooperate in inducing the activation of fibroblasts and their differentiation into myofibroblasts in SSc patients [38]. Thus, understanding the microvascular environment of target organs in SSc is of primary importance.

## 4. Angiogenic Monocytes

It is suggested that the major contribution of the bone marrow to angiogenic processes may come from progenitors of the periendothelial vascular mural cells [39]. Endothelial differentiation of monocyte-derived multipotential cells (MOMCs) can occur with angiogenic stimuli and result in the formation of mature endothelial cell tubules in Matrigel cultures [40]**. **Pericytes establish morphologic interactions with transmigrating leukocytes, mainly monocytes (macrophages) [31]. During angiogenesis, macrophages contribute to the dissociation and detachment of pericytes from the endothelial cell. Pericytes can act as antigen-presenting cells and can behave as macrophages; they also can show plasticity with potential to become myofibroblasts [31]. Thus, understanding the role of the interaction of circulating angiogenic monocyte and resident pericyte in SSc microvasculature has important implications. It is possible that this interaction is of primary importance for linking the inflammatory aspect of the disease to the vascular abnormalities.

Stromal cell-derived factor-1 (SDF-1) and its receptor (CXCR4) system is a component of the microvascular environment which is extremely important for new vessel formation. SDF-1 released by endothelial cells creates a gradient dictating directional response of endothelial cells expressing CXCR4 [41]. Skin biopsies in early disease of both SSc subtypes show a strong positive pattern of SDF-1 and its receptor CXCR4 in the endothelial cells and pericytes of microvessels, attesting to an attempted reparative process [42]. Of interest, in diffuse SSc, these skin biopsies also showed dense mononuclear cells in the perivascular infiltrate, possibly suggesting a role of the monocytes in a more fibrotic phenotype. The staining for CXCR4 was weak in the late (sclerotic or atrophic) phases in both SSc subsets [42]. Another study of 40 SSc patients demonstrated higher serum levels of VEGF, PDGF, and increased concentration of SDF-1, particularly in the diffuse subset. In this same study population circulating CXCR4+ circulating progenitor cells coexpressing monocytic and endothelial cells positively correlated to the severity score, modified Rodnan skin score, and pulmonary involvement [43]. Taken in sum, these results suggest that overall disease activity correlates to the markers of activity in the microvascular environment. 

It has recently been suggested that the actual angiogenic cell type recruited to the site of tissue injury and incorporated into a newly formed vessel is a monocyte [44]. Activated circulating monocytes have also been reported in SSc patients, supporting a potential role of these cells in disease pathogenesis [45]. Gene expression profiling of peripheral blood monocytes from SSc patients suggest that type I interferon may play a key role in the activation of monocytes in this disease [46]. If during the course of the disease, the mechanism of angiogenesis is impaired, the proangiogenic factors in the microvascular environment may serve to recruit proangiogenic monocytes which, with pericytes, result in overactivity of a myofibroblast phenotype. In a preliminary study, there were no significant differences in the expression of circulating monocyte surface molecules involved with cell transformation, function, or migration presumed to give rise to fibrocytes, in 8 patients with limited SSc [47]. It is possible that the role of the angiogenic monocyte may be greater in the diffuse subset of SSc and have prognostic implications.

## 5. Implications of the Vascular Microenvironment on Treatment

An evolving understanding of the vascular microenvironment in SSc may allow directed treatment. Therapeutics that modulate the phenotype of reparative cells can offer new opportunities for SSc treatment [48]. In particular, multipotential MSCs have attracted interest because of low acute toxicity and their availability [49]. The potential of human MOMCs which can proliferate and differentiate along the endothelial lineage in a specific permissive environment also may represent an autologous transplantable cell source for therapeutic neovasculogenesis [40]. In early SSc, prevention of vascular senescence may be most important. N-acetyl-cysteine (NAC), a chemopreventive antiangiogenic and antiapoptotic drug has been suggested to modulate parameters associated with endothelial cell aging [50]. Pilot data suggests that the statin class of medications may be beneficial in treating vascular manifestations of SSc, through an increase in angiogenic factors and reduction of vascular endothelial activation/injury markers (*P* < .01 for all comparisons) [51]. However, this treatment did not correct the defect in EPC recruitment. Cyclophosphamide, which remains the current gold standard for treatment of interstitial lung disease, is known to mobilize EPC [52]. Nutraceutical-based mobilization of EPC is an area of interest in the biomedical field, and has not yet been reported in SSc [53]. 

For the fibrotic aspect of SSc, the small molecule tyrosine kinase inhibitor imatinib and related drugs, such as dasatinib and nilotinib, which simultaneously target two of the major profibrotic pathways, TGF-beta- and PDGF-signaling are being studied [54]. The effect of these drugs on the microvascular environment, and their efficacy and tolerability in SSc patients are not yet known. Other anti-TGF-beta therapies are also in development and may have a major impact in systemic sclerosis. However, considerable concern regarding safety is needed given its pro- and antiangiogenic effects at different concentrations [55]. IFN inhibitors are also under investigation for treatment of SSc, though modulation of interferon may be most effective in the diffuse subset, in which there is a higher perivascular monocyte infiltrate [56]. Specifically, therapies that inhibit transdifferentiation of other cell types, such as pericytes and angiogenic monocytes into fibroblasts and myofibroblasts hold promise [57].

## 6. Conclusion

A predisposition to vascular senescence is probable in SSc and the pathogenesis may arise from a subsequent defect in vasculogenesis (possibly due to abnormal bone marrow function) and/or angiogenesis (perhaps due to pericyte and angiogenic monocytes) followed by overactivity of activated fibroblasts and myofibroblasts. Understanding the role of the vascular microenvironment will be critical to development of directed therapeutics.

Early SSc may be most amenable to treatments that decrease vascular senescence and increase EPC mobilization. Surprisingly, diffuse cutaneous SSc may be more responsive to therapeutics, which modulate pericyte and angiogenic monocyte differentiation into activated fibroblasts and myofibroblasts. The difficulty with therapeutics which modulate growth factor and chemokines is that locally varying levels of these substances are necessary for regulation of migration, proliferation, cell-cell interactions, differentiation, and extracellular matrix deposition [31]. Nonetheless, an improved understanding of the principle regulatory mechanisms of angiogenesis in SSc has profound potential therapeutic value. It is exciting to think that through understanding of the microvascular environment in SSc, that subsequent restoration of proper angiogenesis in SSc could limit fibrotic damage.
